# Bodily distress syndrome: A new diagnosis for functional disorders in primary care?

**DOI:** 10.1186/s12875-015-0393-8

**Published:** 2015-12-15

**Authors:** Anna Budtz-Lilly, Andreas Schröder, Mette Trøllund Rask, Per Fink, Mogens Vestergaard, Marianne Rosendal

**Affiliations:** Research Unit for General Practice, Department of Public Health, Aarhus University, Bartholins Allé 2, 8000 Aarhus C, Denmark; Research Clinic for Functional Disorders and Psychosomatics, Aarhus University Hospital, Aarhus, Denmark

**Keywords:** Bodily distress syndrome, Functional disorders, General practice, Diagnosis, Diagnostic utility, Diagnostic validity, Medically unexplained symptoms

## Abstract

**Background:**

Conceptualisation and classification of functional disorders appear highly inconsistent in the health-care system, particularly in primary care. Numerous terms and overlapping diagnostic criteria are prevalent of which many are considered stigmatising by general practitioners and patients. The lack of a clear concept challenges the general practitioner’s decision-making when a diagnosis or a treatment approach must be selected for a patient with a functional disorder. This calls for improvements of the diagnostic categories. Intense debate has risen in connection with the release of the fifth version of the ‘Diagnostic and Statistical Manual of Mental Disorders’ and the current revision of the ‘International Statistical Classification of Diseases and Related Health Problems’. We aim to discuss a new evidence based diagnostic proposal, bodily distress syndrome, which holds the potential to change our current approach to functional disorders in primary care. A special focus will be directed towards the validity and utility criteria recommended for diagnostic categorisation.

**Discussion:**

A growing body of evidence suggests that the numerous diagnoses for functional disorders listed in the current classifications belong to one family of closely related disorders. We name the underlying phenomenon ‘bodily distress’; it manifests as patterns of multiple and disturbing bodily sensations. Bodily distress syndrome is a diagnostic category with specific criteria covering this illness phenomenon. The category has been explored through empirical studies, which in combination provide a sound basis for determining a symptom profile, the diagnostic stability and the boundaries of the condition. However, as bodily distress syndrome embraces only the most common symptom patterns, patients with few but impairing symptoms are not captured. Furthermore, the current lack of treatment options may also influence the acceptance of the proposed diagnosis.

**Summary:**

Bodily distress syndrome is a diagnostic category with notable validity according to empirical studies. Nevertheless, knowledge is sparse on the utility in primary care. Future intervention studies should investigate the translation of bodily distress syndrome into clinical practice. A particular focus should be directed towards the acceptability among general practitioners and patients. Most importantly, it should be investigated whether the new category may provide the basis for better treatment and improved clinical outcome.

## Background

Many primary-care patients complain of symptoms which cannot be attributed to any conventionally defined medical disease or mental disorder [1–3]. Nevertheless, the conceptualisation and classification of this phenomenon appear to be highly inconsistent, particularly from a primary-care perspective [4].

Numerous terms have been used for symptoms and disorders without a medical diagnosis, e.g. medically unexplained symptoms (MUS), functional symptoms, functional somatic syndromes, central sensitivity syndromes and somatoform disorders. In this paper, we will use the term ‘functional disorders’ as an overarching descriptive term embracing all these differently labelled conditions [5].

From a primary care perspective, there is a serious need for a unifying diagnostic category for functional disorders which should be both evidence based and applicable in the primary care setting. This paper discusses the classification of functional disorders in primary care and focuses on a new diagnostic concept and category of moderate-to-severe functional disorders; bodily distress syndrome (BDS). As we will show, this diagnosis is based on an increasing amount of evidence and has the potential to embrace the numerous conditions and syndromes currently being the source of ongoing debate and controversy in this field. Hence, BDS may provide the needed improvement in classification of functional disorders.

Intense debate fuelled by an increasing demand for improved diagnostic categories of functional disorders were raging during the time leading up to and right after the release of the Diagnostic and Statistical Manual of Mental Disorders, Fifth Edition (DSM-5) [6], and discussions are still ongoing now also in relation to the revision of the International Classification of Diseases (ICD-10) [7]. Without a well-defined concept and general agreement on specific diagnostic categories, many patients with functional disorders remain undetected and are not offered adequate treatment [8]. This shortcoming exists although these conditions are frequent; in the more severe forms, the conditions are even persistent and associated with significant disability, and high societal and health-care costs [3, 9–17].

When patients present with functional disorders in primary care, several circumstances prevent adequate patient management. First, some general practitioners (GP) and patients are sceptical about the concept of functional disorders [18]. Second, there are tremendous variations (3–33 %) in the awareness of the phenomenon among GPs [19, 20]. Furthermore, neither the commonly used term MUS nor the frequently used research method of simply counting symptoms to identify functional disorders are based on diagnostic criteria, and symptom counts have been shown to be unreliable in clinical practice because of the poor sensitivity and specificity of this approach [21].

The present diagnostic ICD-10 classification [7] includes numerous overlapping diagnoses within somatic and psychiatric specialties [22–31], e.g. irritable bowel syndrome, fibromyalgia and somatoform disorders. This diagnostic overlap challenges the GP’s decision-making: Which diagnosis and which referral programme out of the many available would be the most suitable for this particular patient? In addition, the somatoform diagnostic categories presented in the psychiatric chapter of ICD-10 are based on consensus and primarily target severe and chronic cases, but these are rarely used in primary care [20, 32]. These diagnoses are considered stigmatising by many GPs and patients [33, 34]. The DSM-5 diagnosis of complex somatic symptom disorder (CSSD) [6], on the other hand, is more inclusive, but it has been criticised for lack of specificity [35–37].

As a consequence of the lack of a consistent and valid illness concept, and a suitable term for the phenomenon, the ailing patient is labelled rather than the medical condition (e.g. the ‘difficult patient’, the ‘frequent attender’ or the ‘heart-sink patient’). By naming and treating these conditions differently from other medical conditions, we may induce patient resistance. Patients seek the same from their GP whether or not their symptoms can be explained by well-defined medical conditions; constructive dialogue, tangible explanations for their symptoms, and treatment and care [38, 39].

BDS was recently introduced as a diagnostic category. BDS is an empirically based diagnostic category of functional disorders, which encompasses most functional somatic syndromes and somatoform disorders [2, 40]. Observational studies of BDS have been performed on data from primary-care patients [2, 40–44], and results from clinical trials have shown that BDS can serve as a feasible diagnosis of functional disorders in specialised health-care settings [45, 46]. BDS has influenced the current diagnostic proposals for the ICD-11 because of the empirical derivation [47]. We aim to present a brief overview of the underlying concept of functional disorders, which will be followed by an exploration of whether BDS may be a valid and useful alternative to the current diagnostic categories. We discuss whether BDS fits the clinical phenomenon behind functional disorders and whether this diagnostic category may be useful and valid in primary care.

## Discussion and conclusions

### The nature of bodily distress

A growing body of evidence suggests that the many different functional somatic syndromes and somatoform disorders listed in the current classifications belong to a family of closely related disorders, or that they are expressions of the same underlying illness phenomenon with various subtypes [2, 8, 40, 43, 48–50]. This illness phenomenon may be described as bodily distress and is characterised by unpleasant and disturbing bodily sensations. The wide range of conditions labelled with different names show striking similarities in symptom clustering [2, 51], aetiology [48], pathophysiological mechanisms [52], patient characteristics [53], treatment response [8] and co-morbidity [40, 54].

Some studies have investigated how the phenomenon of bodily distress presents. A part of these studies have the major methodological advantage of being based on exploratory approaches and not rely on existing definitions. Across several independent studies from different countries, similar symptom groups have been identified; these represent gastrointestinal symptoms, musculoskeletal symptoms, cardiopulmonary symptoms, neurological/ general symptoms and urogenital symptoms [2, 43, 51, 55–59].

**Fig. 1:**
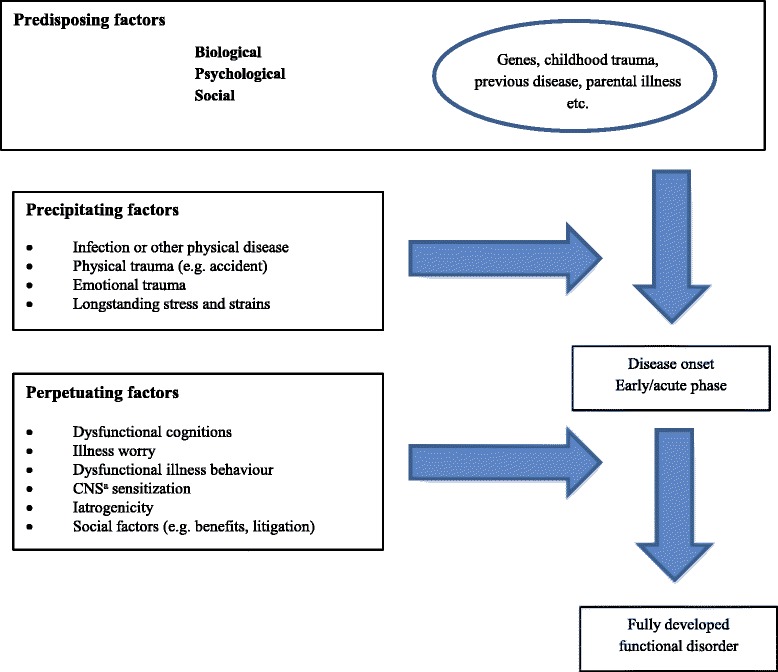
Etiopathogenesis of functional disorders. ^a^ CNS = central nervous system. Modified after Schröder and Dimsdale (2014)

**Fig. 2:**
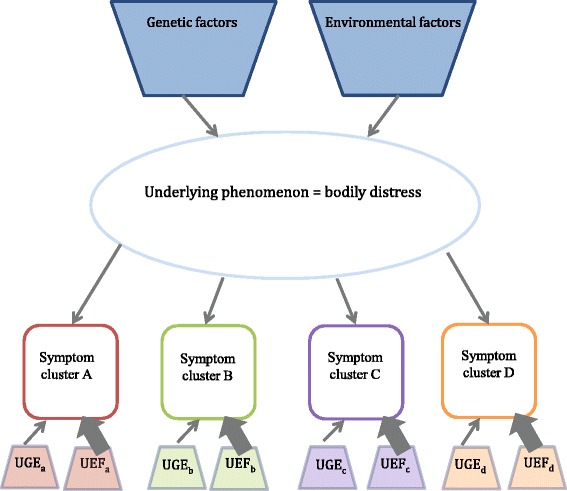
Model of underlying structure for shared predisposing genetic and environmental factors and co-morbidities. The specific syndromes appear primarily to be due to particular environmental influences , e.g. the development of irritable bowel syndrome (IBS) subsequent to a bacterial gastrointestinal infection. A-D are specific symptom clusters, e.g. cardiopulmonary, gastrointestinal, etc. UGF = unique genetic factors, UEF = unique environmental factors

Epidemiological research suggests that the differently labelled conditions share a multifactorial aetiology comprising interacting biological, psychological and environmental factors [48, 60–62]. In other words, the conditions may best be understood as a result of pathophysiological responses to prolonged or severe mental and/or physical stress in genetically susceptible individuals [8, 25, 60, 63–65]. It is still unclear whether perpetuating factors, such as symptom catastrophising and maladaptive coping, are primary or secondary phenomena (see Fig. 1). Some evidence suggests that both genetic and environmental factors are correlated with bodily distress; some are shared (and correlate with the overall phenomenon), while others may be unique for each subcategory of bodily distress [48, 66] and appear to be primarily due to the environmental influences experienced by each individual (see Fig. 2) [48, 67, 68]. Studies have shown that 30-50 % of the patients with differently labelled functional disorders present with concurrent mental disorders, particularly with depression and anxiety [30, 40, 69]. Most studies on comorbidity have been designed as cross-sectional studies and do not allow for any causal interpretation between bodily distress, anxiety and depression. Nevertheless, several studies suggest that the genetic predisposition to develop bodily distress is different from that of developing mental disorders [48, 53, 70, 71]. An increasing volume of evidence indicates that biomedical and neuropsychological processes initiate and maintain bodily distress. Altered autonomic balance [63, 72], stress-axis dysfunction [73–75], sensitised nervous system [76, 77] and activated inflammatory response [78] are all pathophysiological mechanisms that are believed to have the potential to produce and maintain physical symptoms. Finally, similar treatment strategies for various syndromes targeting perpetuating factors appear to be effective in terms of symptom relief and improved functioning. These include exercise, psychological treatment, information and structured care [25, 46, 79–81]. There is some evidence from longitudinal studies that many patients with one functional disorder develop symptoms of other functional disorders, i.e. there appears to be a high degree of mobility between different functional disorders over time [42, 82, 83]. Although we do not know the details of the aetiology and pathogenesis, evidence points strongly towards an underlying phenomenon of bodily distress. A diagnostic category based on this concept may improve the clinical diagnostics and provide a basis for the needed explanatory models.

### The diagnostic category of BDS

The diagnostic category of BDS is based on the symptom groups pertaining to bodily distress and introduces symptom pattern recognition as a core element of the diagnostic criteria. This signifies that the clinician will have to take the same approach as s/he would have done in order to diagnose disease such as arthritis, appendicitis, ischemic heart disease, depression or lupus, i.e. begin with a symptom and then inquire about other symptoms known to be associated with the disease pattern [84]. Patients with BDS present with a specific symptom pattern of bodily distress, which is believed to be associated with pathophysiological disturbances. The symptom pattern is reliably identified by the presence of multiple symptoms within specific symptom groups [2, 40]. The characteristic symptom pattern, combined with a time frame and impairment status, forms the clinical diagnosis of BDS (see Table 1). The diagnostic category comprises a multi-organ type and four single-organ subtypes, and it does not require behavioural or psychopathological features. The identification of BDS is made solely through observation of clinical features, but research to identify pathophysiological markers is now being conducted.

**Table 1:** Diagnostic criteria for BDS

1) ≥ 3 symptoms from at least one of the following groups: • Cardiopulmonary/autonomic arousal:
Palpitations /heart pounding, precordial discomfort, breathlessness without exertion, hyperventilation, hot or cold sweats, dry mouth • Gastrointestinal arousal:
• Abdominal pains, frequent loose bowel movements, feeling bloated/full of gas/distended, regurgitations, diarrhea, nausea, burning sensation in chest or epigastrium
• Musculoskeletal tension:
• Pains in arms or legs, muscular aches or pains, pains in the joints, feelings of paresis or localized weakness, back ache, pain moving from one place to another, unpleasant numbness or tingling sensations
• General symptoms:
Concentration difficulties, impairment of memory, excessive fatigue, headache, dizziness.
2) The patient has been disabled by the symptoms (i.e. daily living is affected) 3) Relevant differential diagnoses have been ruled out • Severity:
Single-organ BDS (mild-moderate): involves one or two of the symptom groups
Multi-organ BDS (severe): involves three or four of the symptom groups

### How does BDS fulfil the principles of diagnostic validity and clinical utility?

In the following, we discuss BDS in relation to the accepted validators of clinical syndromes proposed by Kendell, and Robins and Guze [85–87], the additional criteria suggested by Fink and Rosendal [3, 88] and the criteria for clinical utility proposed by Kendell and First et al. [85, 89] (see Table 2).

**Table 2:** Validators and utility of clinical syndromes, as well as established evidence regarding BDS

Validator	Scientific method	Study
Identify and describe the syndrome	‘Clinical intuition’ or cluster analyses	Fink et al. (2007) [2] Budtz-Lilly et al. (2015) [43]
Demonstrate boundaries between related syndromes and from normality	Statistical methods, e.g. latent class analysis	Fink et al. (2007) [2] Budtz-Lilly et al. (2015) [43]
Establish a distinct course or outcome	Follow-up studies	Budtz-Lilly et al. (2015) [42] Rask et al. (2015) [44]
Establish a distinct treatment response	Therapeutic trials	Fjordback et al. (2013)^a^ [45] Schröder et al. (2012)^a^ [46]
Establish that the syndrome ‘breeds true’	Family studies	No studies found
Identify biological correlates	Demonstrate the association with abnormalities of anatomical, biochemical or molecular character	No studies found
Additional validator		Study
The patients must be sampled from representative populations		Fink et al. (2007) [2] Budtz-Lilly et al. (2015) [42] Budtz-Lilly et al. (2015) [43] Rask et al. (2015) [44]
Results should be confirmed in cross-validation studies		Budtz-Lilly et al. (2015) [43]
Patients must be assessed by an appropriate method		Fink et al. (2007) [2]
Clinical utility		Study
Is it used?		No studies found
Is it acceptable to users?		Lam et al. (2013) [84]
Is it easy to use?		No studies found
Is it used correctly?		No studies found
Does it improve clinical outcome?		No studies found
Does it enhance communication?		
with patients		No studies found^b^
across medical specialties		No studies found
Does it assist in conceptualising?		Lam et al. (2013) [84]

^a^Specialised setting

^b^Applies explanatory models

### The diagnostic validity and BDS

In favour of the validity of BDS is the fact that the clinical description of the BDS symptom profiles originates from principal component analyses analysis based on data from a large study of 978 patients from internal medical and neurological departments and from primary care. The sample consisted of patients consecutively referred to a neurological department (*n* = 120) and an internal medical department (*n* = 157) during a three-month period. From primary care consecutive patients consulting 38 GPs on a new illness problem (*n* = 701) were included. All participating patients were diagnostically interviewed using the Schedules for Clinical Assessment in Neuropsychiatry (SCAN) [90]. This approach entailed that not only a predefined list of symptoms were assessed, but the 76 physical symptoms of the physical health chapter of SCAN were explored. Results from principal component factor analyses identified three symptom groups across patients; a cardiopulmonary group (including autonomic symptoms), a gastrointestinal group and a musculoskeletal group (the fourth group of general symptoms was introduced as described below). However, BDS includes only the most common symptom patterns of bodily distress, whereas, for example, genito-urinary symptoms are not included. In addition, patients with one symptom or very few, but significantly impairing, symptoms will not fulfil the criteria for BDS. Consequently, a group of patients suffering considerably from single symptoms or multiple symptoms not belonging to the described groups will still find themselves between diagnostic categories. As a consequence of the methods used, only fairly prevalent symptom groups were detected, while rare conditions were not identified. Much larger populations are required in order to identify infrequent conditions.

The boundaries of BDS, i.e. the distinction between patients with and without BDS, have been identified in a study by Fink et al. using latent class analysis: The initial analysis did not identify any boundaries as the patients could not be divided into distinct groups. In addition, the results showed that many patients presented with unspecific general symptoms like fatigue and dizziness. Adding a fourth symptom group (consisting of five general unspecific symptoms) to the latent class analysis resulted in the delineation of the BDS construct. Now BDS demonstrated ability to discriminate between ‘no BDS’, ‘single-organ BDS’ and ‘multi-organ BDS’. In other words, patients were divided into three distinct groups as a result of the statistical analyses. Patients with ‘multi-organ BDS’ presented with various symptoms originating from several bodily systems, whereas patients with ‘single-organ BDS’ presented symptoms from one or two bodily systems. ‘Single-organ BDS’ was characterized by the bodily systems involved, i.e. gastrointestinal, cardiopulmonary, musculoskeletal or general symptoms [2].

The typical course of BDS has been described in two follow-up studies [42, 44]. Results from primary care indicate that the condition is rather persistent and that patients with BDS are generally at high risk of poor outcome. Persistency was measured during two years of follow-up, and the results showed that more than half of the patients with BDS at baseline still met the criteria for BDS at follow-up [42]. In comparison, studies on functional somatic syndromes have shown a two-year persistency of chronic widespread pain of 35 % (community population) and a 12-month persistency of chronic fatigue syndrome of 70 % (primary-care population) [91, 92]. Furthermore, a primary-care study with 10-year follow-up showed that BDS was associated with an increased risk of dropping out from the labour market and of receiving public disability benefits [44]. However, a more advanced classification may further specify illness course (episodic, chronic with increasing impairment, chronic with stable impairment, etc.

Two therapeutic trials have been completed [45, 46], and others are currently being conducted on patients with multi-organ BDS. The trials investigate the effect of specialised treatments such as cognitive behavioural therapy, acceptance and commitment therapy, mindfulness-based stress reduction and antidepressants [93]. However, therapeutic trials of patients with single-organ BDS remain to be conducted in a primary-care setting. Studies on family prevalence and potential associations with pathophysiological abnormalities are also lacking in order to further estimate the validity of BDS as a diagnostic concept.

### The additional validity criteria and BDS

The population used for developing the BDS diagnosis is partly representative of primary care as the BDS criteria were based on empirical data from both primary- and secondary-care populations. The data was adequately collected, and the assessment was made in accordance with the procedure for diagnostic SCAN interviews. Regarding a cross-validation of the diagnosis, it is worth mentioning that symptom groups and patient severity groups similar to those of BDS have been identified in several studies [49–51, 55–58, 94]. In addition, the BDS construct has been confirmed in a primary-care population (*n* = 2480) by use of factor and latent class analyses. [43]. However, further studies conducted in different settings and countries are needed in order to consolidate these tentative findings. Furthermore, it has been tested whether patients with six specific functional somatic syndromes (chronic fatigue syndrome, fibromyalgia, irritable bowel syndrome, non-cardiac chest pain, hyperventilation syndrome and pain syndrome) and the DSM-IV somatoform disorders characterised by physical symptoms also met the criteria for BDS. The overall diagnostic agreement between BDS and the investigated diagnoses was 95 % (95 % CI: 93.1–96.0, kappa 0.86, *p* < 0.0001) [40]. These findings indicate that BDS may capture the different diagnoses that appear across the different chapters of the ICD-10 and the somatoform disorders classified according to the (now outdated) DSM-IV.

### The clinical utility and BDS

According to Kendell, a diagnostic category possesses utility when it provides non-trivial information about prognosis, treatment outcomes, and/or testable propositions about biological and social correlates (aetiology) [85]. First et al. proposed a more detailed definition of clinical utility as the extent to which a diagnostic classification assists the clinical decision-makers in conceptualising and communicating clinical information, using the diagnostic categories, choosing effective interventions and predicting future management needs [89]. The clinical utility of a diagnostic category may be assessed by posing the following questions: Is it used? Is it acceptable to users? Is it easy to use? Is it used correctly? Does it improve the clinical outcome? (See Table 2).

So far, it has not been described to what extent BDS is (would be) used in everyday general practice. This is partly because BDS is a new diagnostic category which has not yet been incorporated in the current diagnostic classification systems, and partly because the construct of BDS has not been investigated in implementation studies. In most countries, the health-care system does not offer sufficient (if any) treatment programmes for patients with functional disorders. This would affect the diagnostic utility of any diagnosis. If no treatment options are available, or a diagnostic category has direct negative consequences for the patient, the GP will obviously be reluctant to use such a diagnosis. The lacking treatment options may also lead to low acceptance of BDS and, ultimately, increased risk of stigmatisation.

The user acceptability of BDS has been explored in focus group interviews about general views on the diagnosis among GPs; these interviews were completed in seven countries in connection with the upcoming primary health-care version of the ICD-11 on mental health. There was general agreement that this disorder does exist, but no clear consensus was reached on the proposed diagnostic criteria [84]. Further rating of user acceptability and translation of BDS into clinical practice is currently being conducted in field trials worldwide under the auspices of the World Health Organisation [84].

Whether BDS is easy to use in general practice remains to be examined. However, the fact that BDS has a clear classification algorithm, and that a diagnostic aid, the BDS checklist, has been developed, is in favour of the feasibility [43]. Also the improved conceptualisation provided through theoretically based hypotheses on aetiology may form the basis for a set of explanatory models which could serve as satisfactory candidates for both physicians and patients [84, 95, 96].

Furthermore, whether BDS is used correctly or, most importantly, whether BDS may lead to an improvement of the clinical outcome for patients with functional disorders remains to be investigated.

### Patient acceptability

Data collected for clinical assessment of patients with multi-organ BDS show a high degree of patient acceptability [97], and multi-organ BDS has been used as an inclusion criterion in several clinical trials with low drop-out rates [45, 46]. It is unknown whether the reported acceptability is generalisable to other patient groups.

### Are psycho-behavioural characteristics a prerequisite for the diagnostic criteria?

It is often discussed whether psycho-behavioural characteristics should form part of the diagnostic criteria for functional disorders [47]. BDS has been shown to include most patients with somatoform disorders, even when psycho-behavioural characteristics are not included in the diagnostic criteria [40]. This may indicate that although behavioural and cognitive factors are associated with pathogenesis of functional disorders [60], they may not be prerequisite as diagnostic criteria.

### Terminology

A useful diagnostic category may improve the clinical communication. In this regard, terminology is closely linked to classification as names convey information and meaning by their etymology, associations and connotations [95]. An international group of experts has recommended using a non-stigmatising term, which should be neutral in terms of aetiology and pathology, for this diagnosis; bodily distress syndrome has been considered an option [5]. The term is meant to be descriptive of patient complaints and yet more constructive than ‘medically unexplained symptoms’, a label that many patients find hard to accept [84]. Yet, the recent terminology discussions have also given rise to some concerns with respect to use of the word ’distress’. In some languages, e.g. English, ‘distress’ is generally associated with a description of emotional states [5, 84, 98] rather than a description of a physically impaired state. The terminology discussion seems to continue, even after the proposition of BDS, which is probably because the meaning of the term may vary depending on language and cultural context.

### Does BDS enhance a non-dualistic approach to functional disorders?

Some authors seem to regard BDS as a diagnosis which does not enhance a non-dualistic clinical approach because other relevant differential diagnoses must first be ruled out in order to diagnose a patient with BDS [47]. Two issues will be addressed regarding this viewpoint. First, it is the symptom pattern and not each individual symptom which should be assessed as ‘functional’ or not. This differs from the classification of the current somatoform disorders in the ICD-10; when a clinician decides to apply one of these diagnoses, each symptom must be assessed as either ‘functional’ or ‘of medical origin’. For a BDS diagnosis, the symptoms and the pattern they form must be considered in combination. The physician must, therefore, inquire about symptoms of BDS to identify a characteristic illness picture in the same way as for the diagnostic work-up of any medical or psychiatric condition. It appears less futile to assess whether the expression of the condition as a whole most likely is caused by a well-defined medical condition because a symptom pattern is more specific than single symptoms; symptoms that in the case of functional disorders are often vague and very common. Second, every time a clinician reaches any diagnosis for a patient several differential diagnoses must first be taken into consideration and a disease hypothesis must be tested. In this sense, BDS is not any different.

## Summary

BDS is a diagnostic category that fulfils several important validators for clinical syndromes. However, the current knowledge on the utility of the diagnosis is sparse, and BDS has, so far, been implemented only in specialised health-care settings. Future intervention studies should investigate the translation of BDS into clinical practice and should direct a special focus on the acceptability of the BDS construct among GPs and patients; whether it is used and, most importantly, whether it seems to guide treatment and improve the clinical outcome for the affected patients. However, in order to evaluate the performance and the required adaptation of the BDS construct, training of the health-care professionals is essential, as it is when any new concept is introduced in clinical practice.

A major advantage of the BDS diagnosis is that it has been explored in empirical studies, including primary-care populations. On the other hand, BDS does not embrace all patients with impairing symptoms; a small group of patients with either rare functional symptom patterns or only very few disabling symptoms (or just one disabling symptom) are not included in the category. In addition, all studies on BDS have been conducted in countries of the Western world. The lacking knowledge of the validity of BDS in the developing countries is a drawback. However, field trials studies are being performed worldwide including primary care populations from Brazil, Mexico, Spain, Pakistan and Hong Kong under the auspices of World Health Organization. Finally, the term itself may be a challenge as it translates poorly into some languages and may contribute to dualism, e.g. the word ‘distress’ may, in some cultural contexts, primarily imply emotional aspects.

Reactions to diagnoses of functional disorders vary broadly and there is a need to find common ground and concept. The BDS concept may offer an evidence-based path for uniformly categorising patients with functional disorders and may help relieve some of the classification confusion caused by the existing numerous overlapping labels. Furthermore, BDS may provide the GP with a diagnosis which is more suitable for the clinical picture seen in general practice and opens up for tangible explanatory models. In combination, this may entail more consistent patient management and may also form the basis for more homogeneous research in prevention and improved treatment strategies; hence BDS holds the potential for better patient outcomes.

## Abbreviations

BDS: Bodily distress syndrome
CSSD: Complex somatic symptom disorders
DSM: Diagnostic and Statistical Manual of Mental Disorders
ICD: International Statistical Classification of Diseases and Related Health Problems
GP: General practitioner
MUS: Medically unexplained symptoms
SCAN: Schedules for Clinical Assessment in Neuropsychiatry
